# Shaping Ethylene Response: The Role of EIN3/EIL1 Transcription Factors

**DOI:** 10.3389/fpls.2019.01030

**Published:** 2019-08-26

**Authors:** Vladislav A. Dolgikh, Evgeniya M. Pukhovaya, Elena V. Zemlyanskaya

**Affiliations:** ^1^Institute of Cytology and Genetics, Siberian Branch of Russian Academy of Sciences, Novosibirsk, Russia; ^2^Department of Natural Sciences, Novosibirsk State University, Novosibirsk, Russia

**Keywords:** ETHYLENE-INSENSITIVE3, ETHYLENE-INSENSITIVE3-LIKE, epigenetic regulation, protein–protein interactions, cross-talk

## Abstract

EIN3/EIL1 transcription factors are the key regulators of ethylene signaling that sustain a variety of plant responses to ethylene. Since ethylene regulates multiple aspects of plant development and stress responses, its signaling outcome needs proper modulation depending on the spatiotemporal and environmental conditions. In this review, we summarize recent advances on the molecular mechanisms that underlie EIN3/EIL1-directed ethylene signaling in Arabidopsis. We focus on the role of EIN3/EIL1 in tuning transcriptional regulation of ethylene response in time and space. Besides, we consider the role of EIN3/EIL1-independent regulation of ethylene signaling.

## Key Components of Ethylene Signaling Pathway

**Figure 1:**
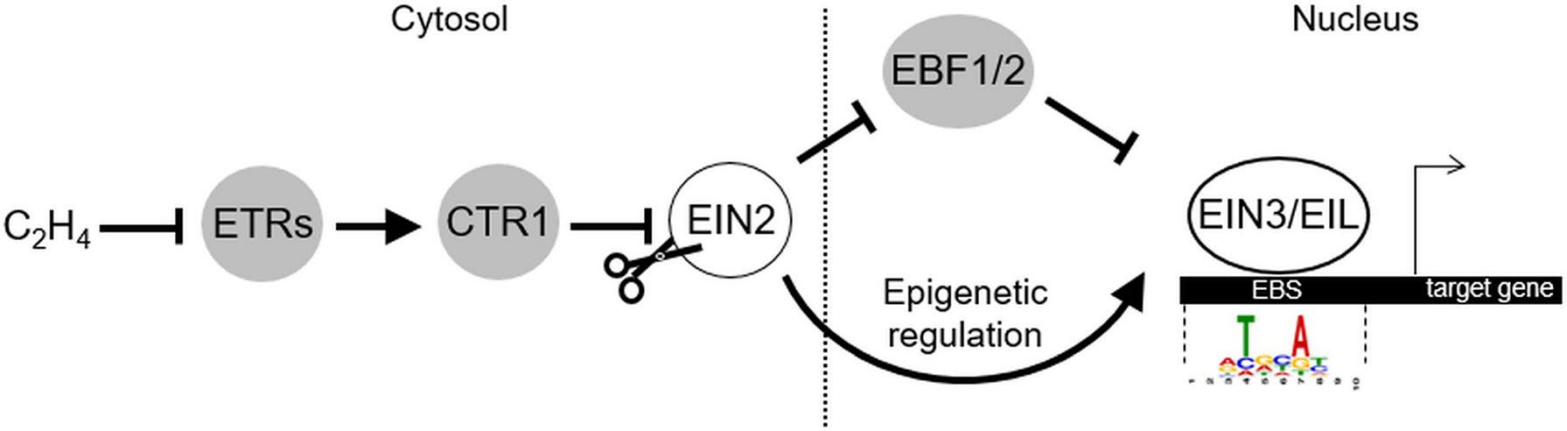
The key components of ethylene signaling pathway. Gray and white circles depict negative and positive regulators of ethylene signaling, correspondingly. Position frequency matrix for Arabidopsis EIN3 binding motif (Chang et al., 2013) was retrieved from CIS-BP database (Weirauch et al., 2014) and visualized using Tomtom tool (http://meme-suite.org/tools/tomtom; Gupta et al. 2007). The model is based on the findings reported previously (Chang, 2016; Hu et al., 2017). The explanations are in the text. EBS, EIN3 binding site.

Plant hormone ethylene coordinates numerous developmental processes (including germination, soil emergence, seedling growth, fruit ripening, senescence, abscission, etc.), as well as diverse biotic and abiotic stress responses (Abeles et al., 2012). Ethylene has also been shown to induce typical morphological changes in dark-grown seedlings (inhibition of hypocotyl and root elongation, radial swelling of hypocotyl, and exaggeration of apical hook) known as “the triple response” (Ecker, 1995). Ethylene is produced from *L*-methionine, which is consequently converted to *S*-adenosyl-*L*-methionine (by SAM-synthetases), 1-aminocyclopropane-1-carboxylic acid (ACC) (by ACC synthases), and ethylene (by ACC oxidases) (reviewed in Booker and DeLong, 2015). Ethylene is perceived by a family of receptors (ETHYLENE RESPONSE 1, ETR1; ETHYLENE RESPONSE SENSOR 1, ERS1; ETR2, ETHYLENE INSENSITIVE 4, EIN4; and ERS2 in Arabidopsis) localized in the endoplasmic reticulum (ER) membrane (reviewed in Lacey and Binder, 2014). Upon binding, ethylene inactivates them and thereby blocks the serine–threonine protein kinase CONSTITUTIVE TRIPLE RESPONSE 1 (CTR1) activity promoting the cleavage of ER-anchored EIN2 protein (reviewed in Chang, 2016; Hu et al., 2017). EIN2 C-terminal domain (EIN2-C) released upon cleavage indirectly triggers EIN3 and EIN3-Like (EIL) transcription factors (TFs) that are considered the key transcriptional regulators of ethylene response (**Figure 1**). Noteworthy, these TFs function as a hub that integrates and processes different cues to “shape” ethylene response in accordance with spatiotemporal and environmental conditions. Below, we will focus on the nuclear events that conduct EIN3/EIL activation and set their functional output.

## Activation of EIN3 and Its Homologs in Response to Ethylene

**Figure 2:**
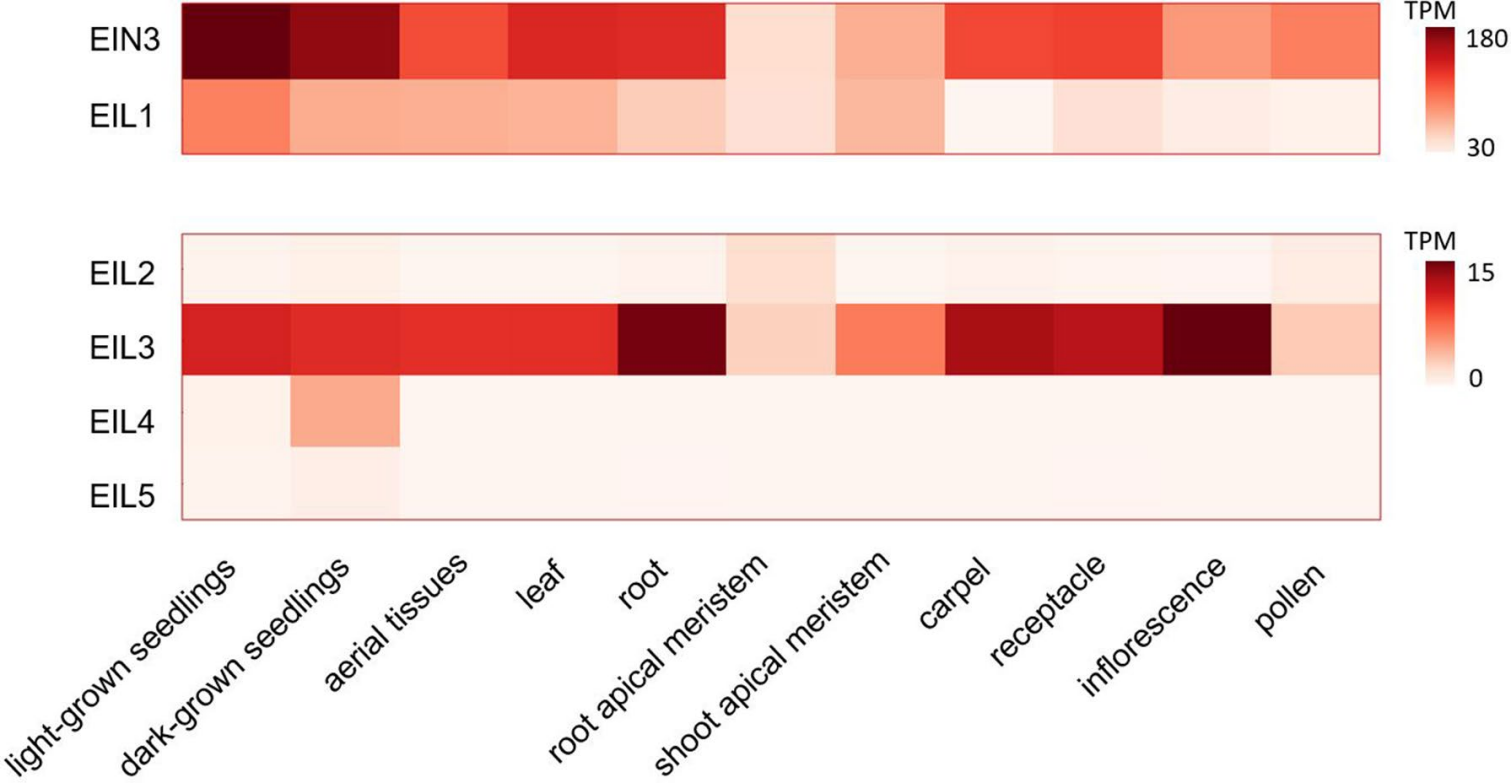
Tissue specificity of *EIL* genes expression. Publicly available datasets on transcriptome profiling of different Arabidopsis tissues (light- and dark-grown seedlings (Rühl et al., 2012; Oh et al., 2014), aerial tissues (Sani et al., 2013), leaf (Wollmann et al., 2012), root (Li et al., 2013), root and shoot apical meristems (Kang et al., 2014; Nozue et al., 2015), carpel (Martínez-Fernández et al., 2014), receptacle (Niederhuth et al., 2013), inflorescence (Gan et al., 2011), pollen (Loraine et al., 2013) were used for visualization. The corresponding expression levels were retrieved from ThaleMine v1.10.4 (https://apps.araport.org/thalemine/; Krishnakumar et al., 2017). TPM, transcripts per million.

EIL is a small family of plant-specific proteins. There are six genes encoding the members of this family in *Arabidopsis thaliana* genome (*EIN3*, *EIL1-5*) (Chao et al., 1997; Guo and Ecker, 2004). They harbor a conservative N-terminal DNA-binding domain with a unique fold structure (Song et al., 2015). EIN3, EIL1, and EIL2 represent functionally homologous proteins involved in the regulation of ethylene-responsive genes (Chao et al., 1997; Solano et al., 1998; Alonso et al., 2003; An et al., 2010). The most closely related EIN3 and EIL1 are considered the major regulators since *ein3 eil1* double mutants show complete ethylene insensitivity in terms of the triple response, pathogen resistance, and the ability to fully suppress *ctr1* mutation (reviewed in Guo and Ecker, 2004; Cho and Yoo, 2015). Two paralogs differentially regulate ethylene response in the seedlings (EIN3) and in adult leaves and stems (EIL1) (An et al., 2010). Yet, a minor, *EIL2* role in the regulation of ethylene response is supported by its capability to complement *ein3* mutation when overexpressed (Chao et al., 1997). In **Figure 2**, we visualized tissue-specific expression levels of *EIL* genes based on publicly available data on transcriptome profiling in different Arabidopsis tissues retrieved from ThaleMine v1.10.4 (https://apps.araport.org/thalemine/; Krishnakumar et al., 2017). Unlike *EIN3* and *EIL1*, *EIL2* transcripts level is low throughout plant tissues; moderate *EIL2* expression is restricted to root apical meristem and pollen (**Figure 2**). Therefore, EIL2 function could be limited to specific spatiotemporal conditions. EIL3/SLIM1 does not function in ethylene pathway but regulates sulfur deficiency response; no defined roles of EIL4 and EIL5 have been reported to date (reviewed in Guo and Ecker, 2004; Wawrzyńska and Sirko, 2014).

**Figure 3:**
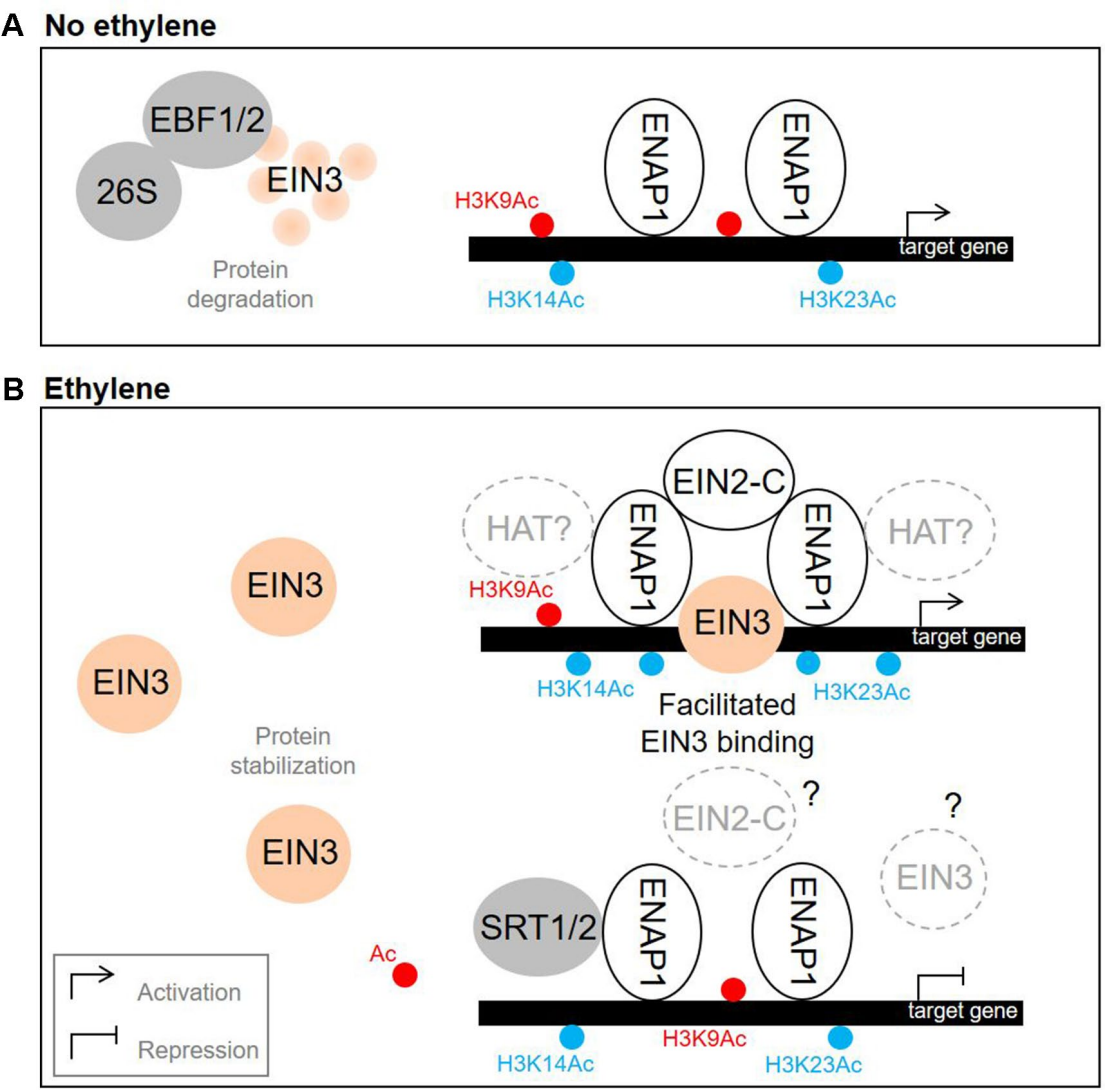
Nuclear events that promote ethylene response. **(A)** Without ethylene, EIN3 undergoes EBF1/2-driven degradation. **(B)** Upon ethylene treatment, EIN3 is stabilized. On one hand, EIN2 C-terminal domain interacts with ENAP1, which results in elevation of H3K14Ac and H3K23Ac levels, facilitated EIN3 binding to the target promoters and activation of gene expression. On the other hand, SRT1 and SRT2 histone deacetylases mediate ethylene-directed transcriptional repression by downregulating the levels of H3K9 acetylation. The models are based on the findings reported previously (Gagne et al., 2004; Li et al., 2015; Merchante et al., 2015; Zhang et al., 2016; Zhang et al., 2017; Zhang et al., 2018a). Gray and white solid circles depict negative and positive regulators of ethylene signaling, correspondingly. EIN3 is depicted in orange, H3K9Ac—in red, H3K14Ac and H3K23Ac—in blue. Dashed circles denote putative regulators (with a question mark inside) and putative regulations (with a question mark outside). HAT, histone acetyltransferase; 26S, 26S proteasome.

EIN3 and EIL1 activation in response to ethylene is the target for complex regulation. EIN3 and EIL1 are short-living proteins that undergo ubiquitination and proteasomal degradation driven by ubiquitin-ligases EIN3 BINDING F-BOX1 (EBF1) and EBF2 (**Figures 1** and **3A**) (Gagne et al., 2004; An et al., 2010). Stabilization of EIN3/EIL1 upon ethylene release plays a pivotal role in triggering ethylene-directed gene expression. Ethylene dampens EBF1/2 levels *via* i) translational repression of *EBF1*/*2* mRNA in the cytosol promoted by EIN2-C (Li et al., 2015; Merchante et al., 2015), and ii) EIN2-dependent proteasomal degradation of EBF1/2 proteins (An et al., 2010) (**Figure 1**). Stabilized EIN3/EIL1 accumulate in the nucleus.

EIN3/EIL1 are predominantly transcriptional activators (Chang et al., 2013; reviewed in Cho and Yoo, 2015). In Arabidopsis, EIN3, EIL1, and EIL2 specifically bind a short DNA sequence referred to as EIN3 binding site (EBS) in gene promoters (**Figure 1**) (Solano et al., 1998; Chang et al., 2013; Song et al., 2015; O’Malley et al., 2016). EIN3 binds its target loci as a homodimer, and the dimerization is DNA independent (Solano et al., 1998; Song et al., 2015). Accordingly, EIN3 demonstrates higher binding affinity to the inverted repeats of EBS compared to the monomeric site in the *in vitro* experiments (Song et al., 2015). EIN3 binding to the targets is facilitated by elevated levels of H3K14 and non-canonical H3K23 histone acetylation both promoted by a EIN2-C-scaffolded histone acetylation complex, which is triggered upon EIN2-C interaction with a histone binding protein EIN2 NUCLEAR ASSOCIATED PROTEIN 1 (ENAP1) (Zhang et al., 2016; Zhang et al., 2017; reviewed in Wang and Qiao, 2019) (**Figure 3**). Since neither EIN2-C nor ENAP1 possess histone acetyltransferase domains, they might recruit other proteins to promote histone modifications. EIN3 is capable of interacting with ENAP1, too, and it is thought to contribute to ethylene-induced elevation of H3K14 and H3K23 acetylation as well (Zhang et al., 2016).

Along with well-known EIN3/EIL1-promoted gene transcriptional activation, ethylene downregulates a considerable set of genes (Chang et al., 2013; Harkey et al., 2018). In a recent work, Zhang et al. (2018a) demonstrated that histone deacetylases SRT1 and SRT2 mediate transcriptional repression in response to ethylene by downregulating the levels of H3K9 acetylation (at least for a particular set of ethylene-repressed genes) (**Figure 3**). Both deacetylases interact with ENAP1, and the function of *SRT2* is *EIN2*- and *EIN3/EIL1*-dependent. The mechanism used to distinguish between the activator and repressor pathways as well as the role of EIN3/EIL1 in SRT1/2-mediated gene repression are still unclear and need further investigations.

## EIN3/EIL1-Regulated Transcriptional Networks

Upon DNA binding, EIN3/EIL1 modulate multiple transcriptional cascades. Ethylene-sensitive EIN3 target genes encoding TFs include *ERF1*, involved in a range of ethylene responses (Solano et al., 1998), *PIF3*, *RSL4*, *ESE1*, and *CBF1/2/3*, the regulators of de-etiolation, root hair development, salt and cold stress responses, correspondingly (Zhang et al., 2011; Shi et al., 2012; Zhong et al., 2012; Feng et al., 2017). To supplement this list, numerous TF-encoding genes comprising representatives of AP2/ERF, WRKY, NAC, and other families were retrieved from whole-genome data on EIN3 binding and ethylene-induced transcriptomes (Chang et al., 2013). Besides, EIN3 directly regulates expression of chlorophyll biosynthesis genes *PORA/B* (Zhong et al., 2009), the pigment-binding proteins *LHC* essential for photosynthesis initiation (Liu et al., 2017), the immune receptor *FLS2* (Boutrot et al., 2010), and the apical hook formation regulator *HLS1* (Lehman et al., 1996; Shen et al., 2016). EIN3/EIL1 affect the pathways of many hormones (Chang et al., 2013), including direct regulation of hormones biosynthesis (e.g., salicylic acid biosynthesis gene *SID2*, Chen et al., 2009), and signaling (e.g., type-A negative regulators of cytokinin signaling *ARR5/7/15*, Shi et al., 2012). To maintain a homeostasis, EIN3 activates a feedback regulatory circuit by inducing transcription of *EBF2* (Konishi and Yanagisawa, 2008) and probably some other negative regulators of ethylene signaling (Chang et al., 2013).

To provide a proper phenotypic outcome upon ethylene release, these transcriptional cascades and the downstream growth control pathways should be tightly coordinated, which is supported by data on the dynamic changes of ethylene-induced transcriptomes in etiolated Arabidopsis seedlings where four distinct transcriptional waves are segregated (Chang et al., 2013). The observed transcription kinetics may be due to distinct mechanisms of transcriptional control, or the heterogeneity of the ethylene response in different tissues (Chang et al., 2013). Transcriptome profiling of Arabidopsis mutants identified large groups of EIN3/EIL1-regulated genes that were co-regulated by the other TFs such as RHD6 (root hair development) and PIFs (light signaling) (Feng et al., 2017; Shi et al., 2018), which implies co-regulation of EIN3/EIL1-triggered transcription by certain developmental and environmental cues. In the following sections, we illustrate that EIN3/EIL1 proteins represent crucial targets for tuning the downstream transcriptional cascades in time and space.

## Tuning Transcriptional Regulation of Ethylene Response

### Epigenetic Regulation of Spatiotemporal Expression of EIN3/EIL1 Target Genes

Climacteric fleshy fruits (the ones that demonstrate a respiratory burst at the beginning of ripening) use ethylene as a ripening signal (McMurchie et al., 1972). Mature fruit produces ethylene in an autocatalytic manner (system II) unlike immature fruit and vegetative tissues where self-inhibitory ethylene production (system I) is implemented. Autocatalytic regulation suggests a positive feedback loop controlling ethylene synthesis. Presumably, the corresponding regulatory circuit includes EIN3 triggered transcriptional cascade that finally activates ethylene biosynthesis genes (*ACSes* and *ACOs*) (Vandenbussche et al., 2012; Lü et al., 2018). To prevent uncontrolled ethylene production, this circuit should be under a tight spatiotemporal regulation.

Epigenetic modifications often promote spatiotemporal regulation of plant hormone responses (reviewed in Yamamuro et al., 2016). In Arabidopsis, a repressive mark H3K27me3 regulates expression of a large number of genes (Lafos et al., 2011). A systematic analysis of epigenome and transcriptome data suggests that climacteric fruits use removal of H3K27me3 to trigger autocatalytic system II ethylene production specifically in the mature fruit (Lü et al., 2018). Accordingly, EIN3 targeted promoters—a part of transcriptional feedback circuit controlling climacteric fruit ripening (*RIN* in tomato, *NAC* in peach and banana)—are associated with the repressive histone mark H3K27me3 in leaf and immature fruit. They become demethylated and therefore accessible only in the ripening fruit tissues. Presumably, this epigenetic mechanism prevents autocatalytic ethylene production in vegetative and immature fruit tissues.

Recently, using a systematic analysis of publicly available ChIP-Seq data on EIN3 binding in Arabidopsis, we have demonstrated that EIN3 direct targets are enriched in a chromatin state 4 according to the classification of Sequeira-Mendes et al. (2014), which is associated with H3K27me3 repressive mark (Zemlyanskaya et al., 2017b). Therefore, H3K27me3-associated epigenetic silencing might be a more general mechanism providing spatiotemporal specificity of ethylene response *via* restriction of EIN3 function.

### Modulation of EIN3/EIL1 Protein Stability

Regulation of EIN3/EIL1 levels *via* the control of the protein stability by EBF1/2 is a pivotal mechanism of EIN3/EIL1 adjustment in ethylene signaling. Simultaneously, it can be affected by environmental factors resulting in a modulation of transcriptional response to ethylene. Plants germinating in the darkness assume a light-regulated developmental program known as skotomorphogenesis, which phenotypically results in rapid hypocotyl elongation, small closed chlorotic cotyledons, and apical hook formation (McNellis and Deng, 1995). EIN3/EIL1 and their target genes (e.g., *HLS1*, *ERF1*, *PIF3*, *PORA/B*) play essential roles in these processes. They contribute in chlorosis and increased apical hook curvature of buried seedlings, induce shortening and thickening of hypocotyl to enhance lifting capacity of the seedling, and finally promote seedlings greening upon light irradiation (Zhong et al., 2009; Zhong et al., 2012; Zhong et al., 2014; Shen et al., 2016).

In the seedlings growing through the soil, EIN3/EIL1 are stabilized by both light signaling and ethylene, which accumulates in response to mechanical pressure. In the former case, E3 ubiquitin ligase CONSTITUTIVE PHOTOMORPHOGENIC 1 (COP1), a central repressor of light signaling, directly targets EBF1/2 for ubiquitination and degradation (Shi et al., 2016a). As seedlings grow toward the surface, light intensity gradually increases. As a result, COP1 activity, which is negatively regulated by photoreceptors (Podolec and Ulm, 2018), gradually decreases attenuating ethylene response.

When the seedling reaches the soil surface, light triggers a dramatic developmental transition known as de-etiolation that leads to immediate termination of ethylene responses. Light-activated photoreceptor phytochrome B (phyB) directly interacts with both EIN3 and EBF1/2 proteins, thereby stimulating robust EIN3 degradation, rapidly turning off ethylene signaling (Shi et al., 2016b; Luo and Shi, 2019).

### Repression of EIN3/EIL1 Transcriptional Activity

In this section, we consider the cross-talk of ethylene signaling pathway with jasmonic acid (JA) and gibberellins (GA) based on an inhibition of EIN3/EIL1 transcriptional activity due to their physical interactions with repressor proteins (**Table 1**). These protein–protein interactions (PPI) rather prevent EIN3/EIL1 binding to DNA than cause changes in protein stability (Zhu et al., 2011; An et al., 2012; Zhang et al., 2014). JA and ethylene synergistically regulate certain aspects of plant development (such as root hair development and inhibition of root growth) and tolerance to necrotrophic fungi. The transcriptional repressors JASMONATE ZIM-DOMAIN (JAZ) are the master regulators that interact with MYC2, MYC3, and MYC4 TFs and negatively control JA signaling (reviewed in Wasternack and Song, 2017). JAZ proteins interact with EIN3/EIL1 and enhance EIN3/EIL1 binding to HDA6, an RPD-type histone deacetylase (Zhu et al., 2011; Zhu and Lee, 2015). The resulting complex inhibits EIN3/EIL1-mediated transcription. Upon JA treatment, JAZ degrades, attenuating HDA6-EIN3/EIL1 association and therefore activating EIN3/EIL1. Therefore, pathogenesis-related genes *ERF1* and *ORA59*, directly regulated by EIN3/EIL1, as well as their downstream target *PDF1.2*, are upregulated in response to JA.

**Table 1:** Protein–protein interactions involved in modulation of EIN3/EIL1 function.

Protein	Organism	Pathway	Function	PPI targets	Interaction output	Reference
***EIN3/EIL1 stability***						
EBF1/2	*Arabidopsis thaliana*	Ethylene signaling	F-box protein	EIN3/EIL1	EIN3/EIL1 degradation	Gagne et al., 2004; An et al., 2010
COP1	*Arabidopsis thaliana*	Light signaling	E3 ubiquitin ligase	EBF1/2	EIN3/EIL1 stabilization	Shi et al., 2016a
phyB	*Arabidopsis thaliana*	Light signaling	Protein binding	EIN3/EIL1, EBF1/2	EIN3 degradation	Shi et al., 2016b
AKIN10	*Arabidopsis thaliana*	Catabolic pathways	PK	EIN3	EIN3 degradation	Kim et al., 2017
***EIN3/EIL1 repression***						
RGA, GAI	*Arabidopsis thaliana*	GA signaling	RP	EIN3/EIL1/2	EIN3/EIL1 repression	An et al., 2012
JAZ1	*Arabidopsis thaliana*	JA signaling	RP	EIN3/EIL1, HDA6	EIN3/EIL1 repression in complex with HDA6	Zhu et al., 2011
MYC2/3/4	*Arabidopsis thaliana*	JA signaling	TF	EIN3/EIL1	EIN3/EIL1 repression	Song et al., 2014; Zhang et al., 2014
EIL3/SLIM	*Arabidopsis thaliana*	Sulfur deficiency response	TF	EIN3	EIL3/SLIM repression	Wawrzyńska and Sirko, 2016
***EIN3/EIL1 cooperation with other TFs***						
RHD6	*Arabidopsis thaliana*	Root hair formation	TF	EIN3	*RSL4* co-activation	Feng et al., 2017
PIF3	*Arabidopsis thaliana*	Light signaling		EIN3	*LHC* co-repression	Liu et al., 2017
CpNAC2	*Carica papaya* L.	Carotenoid biosynthesis	TF	CpEIN3a	*CpPDS4* and *CpCHY-b* co-activation	Fu et al., 2017
FIT	*Arabidopsis thaliana*	Iron acquisition pathway	TF	EIN3/EIL1	FIT stabilization	Lingam et al., 2011
MED25	*Arabidopsis thaliana*	N/A	Mediator subunit	EIN3/EIL1	FIT activation	Yang et al., 2014
ARR1	*Arabidopsis thaliana*	Cytokinin signaling	TF	EIN3	ARR1 activation	Yan et al., 2017

PPI, protein–protein interaction; JA, jasmonic acid; GA, gibberellins; TF, transcription factor; RP, repressor protein; PK, protein kinase.

At the same time, MYC2, MYC3, and MYC4 transcriptional regulators of JA signaling interact with EIN3/EIL1, inhibiting their function (Song et al., 2014; Zhang et al., 2014). Thus, *ERF1*, *ORA59*, and *PDF1.2* genes are upregulated in *myc2* mutants. This inhibitory mechanism underlies ethylene-JA antagonism. Particularly, JA-directed abolishment of ethylene-promoted apical hook formation proceeds *via* MYC2-mediated attenuation of *HOOKLESS1* (*HLS1*) expression, which is the key regulator of hook development and a direct EIN3/EIL1 target (Lehman et al., 1996; An et al., 2012; Song et al., 2014; Zhang et al., 2014). Additionally, MYC2 targets *EBF1*, inducing its expression and therefore promoting EIN3/EIL1 degradation (Zhang et al., 2014). Noteworthy, the inhibitory effect in the EIN3–MYC2 complex is reciprocal: the interaction suppresses MYC2 activity as well and thereby ethylene attenuates JA-regulated plant defense response against insect attack (Song et al., 2014). Similarly, EIN3 plays an inhibitory role in sulfur deficiency response, forming heterodimers with EIL3/SLIM1 TF and preventing its target gene recognition by EIL3/SLIM1 (Wawrzyńska and Sirko, 2016).

Just as in the case of JA-ethylene synergy, GA enhances apical hook curvature at least partially *via* a release of EIN3/EIL1 from repressor proteins. DELLA proteins are the main transcriptional repressors of GA responses (Sun and Gubler, 2004). Two members of this family (RGA and GAI) are capable of associating with EIN3/EIL1 DNA-binding domain and inhibiting EIN3/EIL1 function (An et al., 2012). In response to GAs, DELLA proteins rapidly degrade, thereby de-repressing EIN3/EIL1-mediated transcription of at least the *HLS1* gene.

### EIN3/EIL1 Cooperate With Other TFs in an Interdependent Manner

EIN3/EIL1’s capability to function cooperatively with the transcriptional regulators of the other signaling pathways provides another possibility to shape spatiotemporal patterns of ethylene response. This cooperation implies the cross-talk of TFs bound to DNA that goes along with the physical interaction of these TFs (**Table 1**). In buried seedlings, the chloroplasts’ development is arrested at the etioplast stage, characterized by an immature arrangement of the inner membranes and pigment molecules (Solymosi and Schoefs, 2010; Jarvis and López-Juez, 2013). EIN3 and PHYTOCHROME INTERACTING FACTOR3 (PIF3), a darkness-stabilized transcriptional regulator of light signaling, form an interdependent module that represses chloroplast development in buried seedlings (Liu et al., 2017). Namely, EIN3 and PIF3 directly interact and bind the promoters of *LIGHT HARVESTING COMPLEX* (*LHC*) genes in a cooperative manner to synergistically suppress their expression. Upon light exposure, the levels of EIN3 and PIF3 decrease, and activation of *LHC* expression triggers chloroplast differentiation.

Interestingly, another TF from PIF family, PIF4, interacts with EIN3 as well (Yazaki et al., 2016), and both TFs target *HLS1*, the key regulator of apical hook development (An et al., 2012; Zhang et al., 2018b). However, EIN3 and PIF4 activate *HLS1* transcription independently (Zhang et al., 2018b).

Cooperative regulation also guides ethylene functioning in root hair development. EIN3 promotes root hair elongation by directly activating *RHD6-LIKE4* (*RSL4*) gene (Feng et al., 2017). Besides, EIN3 physically interacts with ROOT HAIR DEFECTIVE6 (RHD6), a major regulator of root hair development that targets *RSL4* as well (Yi et al., 2010; Feng et al., 2017). Both EIN3 and RHD6 co-activate *RSL4* more efficiently than either of them alone (Feng et al., 2017). The role of EIN3–RHD6 cooperative action is most likely not limited to *RSL4* regulation, but rather covers a quite extensive set of genes and contributes to ethylene-promoted root hair initiation as well (Feng et al., 2017). Similarly, in papaya, EIN3 homolog CpEIN3a interacts with CpNAC2, and both TFs directly activate the transcription of carotenoid biosynthesis-related genes *CpPDS4* and *CpCHY-b* expressed during fruit ripening (Fu et al., 2017). Both TFs possess a combinatory effect on the regulation of their targets.

Besides, EIN3/EIL1 are capable of binding gene promoters and affecting gene expression indirectly *via* physical interactions with other TFs and modulation of their activity (**Table 1**). Increase of auxin biosynthesis in the root tip epidermis *via* upregulation of *TRYPTOPHAN AMINOTRANSFERASE OF ARABIDOPSIS 1* (*TAA1*) plays a pivotal role in ethylene-induced inhibition of root growth (Vaseva et al., 2018). EIN3 targets *TAA1* promoter through a “piggyback” interaction with RESPONSE REGULATOR 1 (ARR1), a transcriptional regulator of cytokinin signaling, thereby enhancing ARR1 transcriptional activity (Yan et al., 2017). Similarly, EIN3/EIL1 interact with FER-LIKE FE DEFICIENCY-INDUCED TRANSCRIPTION FACTOR (FIT), a central regulator of Fe acquisition in roots, activating FIT abundance (Lingam et al., 2011). Moreover, EIN3/EIL1 bridges FIT to the transcriptional Mediator complex to recruit RNA-pol and promote the regulation of iron homeostasis (Yang et al., 2014).

## EIN3/EIL1-Independent Ethylene Signaling

There is growing evidence that despite their essential role, EIN3/EIL1 TFs are not indispensable components of ethylene response. Thus, kinetic studies distinguish two phases of ethylene-induced growth inhibition of the hypocotyl in etiolated Arabidopsis seedlings: a transient phase I (up to 2 h) and a sustained phase II (Binder et al., 2004; Chang et al., 2013). Both phases require EIN2 function, while only the second requires EIN3/EIL1 (Binder et al., 2004). Intriguingly, unlike etiolated seedlings, light-grown *ein3 eil1* double mutants do not demonstrate the total loss of long-term ethylene responses (Harkey et al., 2018). Moreover, osmotic stress-induced cell cycle arrest in leaf primordia that coincides with enhanced activation of the ethylene signal is EIN3 independent (Skirycz et al., 2011). These observations favor the existence of an alternative pathway. One possible candidate to promote such regulation is a PAM domain-containing protein EER5. It negatively regulates ethylene signaling during hypocotyl elongation in etiolated seedlings regardless of EIN3 by promoting downregulation of a gene subset upon ethylene treatment. In addition, it physically interacts with EIN2-C (Christians et al., 2008). EER5 regulates magnitude of ethylene response *via* perception of ERS1 signal (Deslauriers et al., 2015).

## Concluding Remarks and Perspectives

Ethylene response is a target for a complex regulation, in which EIN3/EIL1 TFs play a crucial role. Recent studies shed light on multiple layers of complexity in tuning EIN3/EIL1 function (including epigenetic gene silencing and modulation of EIN3/EIL1 stability and activity *via* PPIs) that facilitate the “shaping” of ethylene response according to spatiotemporal and environmental conditions. At the same time, these findings open up new perspectives for further research. Growing evidence of the important role that epigenetic landscape plays in EIN3/EIL1 functioning requires its more detailed characterization. Particularly, the contribution of distinct epigenetic modifications as well as ENAP1 patterns in modulation of EIN3/EIL1 function is of interest. In view of interdependent cooperation of EIN3/EIL1 with some TFs described recently, the detailed analysis of nucleotide context surrounding EIN3 binding sites requires more attention, and genome-wide research appears helpful both to generalize resent findings and to predict new connections. Moreover, it would be interesting to clarify the role of epigenetic regulation and PPIs in suppression of gene expression upon ethylene treatment.

Yet, despite their essential role, EIN3/EIL1 are not indispensable regulators of ethylene response. To couple the molecular events and phenotypic responses more precisely, EIL2 function in ethylene signaling and EIN3/EIL independent pathways are to be elucidated.

## Author Contributions

VAD and EMP performed the literature search and drafted the paper. VAD performed the analysis of the transcriptome datasets. EVZ revised and edited the manuscript. All authors read and approved the final manuscript.

## Funding

The work was funded by the Russian Foundation for Basic Research through grant № 18-29-13040. Meta-analysis of the transcriptome datasets was done in the frame of the project supported by the Russian Foundation for Basic Research and the government of Novosibirsk region through grant № 18-44-540039.

## Conflict of Interest Statement

The authors declare that the research was conducted in the absence of any commercial or financial relationships that could be construed as a potential conflict of interest.
